# Cervical kyphosis in patients with Lenke type 1 adolescent idiopathic scoliosis: the prediction of thoracic inlet angle

**DOI:** 10.1186/s12891-017-1590-5

**Published:** 2017-05-25

**Authors:** Ce Zhu, Xi Yang, Bangjian Zhou, Lei Wang, Chunguang Zhou, Tingxian Ling, Limin Liu, Yueming Song

**Affiliations:** Department of Orthopedics Surgery, West China Hospital, Sichuan University, No. 37 GuoXue Road, Chengdu, Sichuan 610041 China

**Keywords:** Adolescent idiopathic scoliosis, Lenke type 1, Cervical kyphosis, Thoracic inlet angle, Correction surgery

## Abstract

**Background:**

Several studies have explored cervical kyphosis (CK) in adolescent idiopathic scoliosis (AIS) patients. However, few studies have evaluated the cervical alignment in these patients according to their coronal curve type. The aim of this study was to analyze the radiological features of cervical sagittal alignment in Lenke 1 AIS patients before and after surgery.

**Methods:**

This is a retrospective study enrolled 50 patients. Preoperative and postoperative standing full-length radiographs (at last follow-up after operation) were used to measure the coronal and sagittal parameters. Main sagittal parameters included C2–C7 angle, thoracic inlet angle (TIA), T1 slope, proximal thoracic kyphosis (PTK, T1-5 kyphosis) and thoracic kyphosis (TK, T5-12 kyphosis).

**Results:**

The TIA of patients with CK was significantly smaller than that of patients with CL (63.0° vs. 76.3°, *p* < 0.05) and the cutoff value was 71°. The TIA of patients with CK after surgery was significantly smaller than that of patients with CL postoperatively (62.5° vs. 74.6°, *p* < 0.05) and the cutoff value was 62°. In patients with postoperative CL, there was a significant increase in their PTK and a reduction in their TK, regardless of preoperative CL or CK. In patients whose CL deteriorated to CK after surgery, both their PTK and TK significantly decreased after surgery.

**Conclusions:**

Patients with TIA less than 71° were more likely to have CK. And patients with TIA less than 62° would lead to the postoperative uncorrected or new onset of CK. The increased PTK after operation could have a beneficial effect on the improvement of CL.

## Background

Adolescent Idiopathic Scoliosis (AIS) is a three-dimensional deformity of the spine. Compared to coronal balance, spinal sagittal balance is more unclear and abstract due to various complex curved appearances in the human lateral view. The relationship between the thoracic spine, lumbar spine and pelvic parameters and the pre- and postoperative changes of them have widely been studied in the sagittal plane [1–3]. However, the correlation between cervical sagittal alignment and global sagittal alignment in AIS patients has not been thoroughly addressed.

As a part of global sagittal alignment, cervical sagittal alignment correlated with health-related quality of life (HRQOL) in AIS patients [4–6]. In addition, cervical kyphosis (CK) may play a large role in the development of cervical myelopathy [7–9]. Therefore, more studies have paid attention to the CK in AIS patients recently. Hilibrand et al. [10] confirmed a significant correlation between the loss of thoracic kyphosis (TK) and CK in AIS patients. Similar relations were also found in some recent studies [11–13]. Thereafter, Legarreta et al. [14] found that both pedicle screws and hybrid instrumentation (hooks and pedicle screws) have a hypokyphotic effect on the thoracic spine, which could lead to the occurrence of CK after the surgery, especially in cases in which the upper instrumented vertebra (UIV) is above the T4 level. However, Yanik et al. [15] indicated that the postoperative CK was correlated with the decreased TK and T1 slope, nor UIV level. Pesenti S et al. [16] considered that T1 slope was a good indicator of postoperative changes for cervical and thoracic parameters.

Cervical alignment might be affected by structural proximal thoracic, thoracic, and/or lumbar scoliosis, but few studies have evaluated the cervical alignment according to their coronal curve types among these studies mentioned above [13, 15]. Thus, we focused on the analysis of the radiological features of cervical alignment in patients affected by structural thoracic curve (Lenke type 1) before and after surgery.

## Methods

This study was a retrospective analysis of 50 consecutive patients with Lenke 1 type AIS who underwent surgery in our department from September 2010 to August 2015. All patients underwent surgical treatment with 1-stage posterior pedicle-screw correction and fusion. The inclusion criteria were: (1) age below 18 years at the time of surgery, (2) a diagnosis of Lenke type 1 adolescent idiopathic scoliosis, (3) a Cobb angle of the thoracic curve more than 40°, (4) no neurological deficit, (5) no previous spine surgery, and (6) at least 1 years of radiographic follow-up with adequate visualization of the cervical spine on pre- and postoperative films.

All surgical procedures were performed by the same surgeon. The UIV was in T2–T5 (T2, *n* = 6; T3, *n* = 28; T4, *n* = 13; T5, *n* = 3) and the lower instrumented vertebra (LIV) was in L1–L4 (L1, *n* = 18; L2, *n* = 16; L3, *n* = 11; L4, *n* = 5). Several surgical maneuvers were used in the operation, including rod-rotation, apical vertebral derotation (by vertebral column manipulation or vertebral coplanar alignment appliance), convex compression and concave distraction. The CD HORIZON M8 or Legacy screw-rod system (Medtronic Sofamor Danek Inc., Memphis, TN) was used for fixation.

Standing full-length posteroanterior and lateral radiographs by the multipurpose Digital R/F System (Sonialvision Safire 17; Shimadzu Corp., Kyoto, Japan) were obtained before and at last follow-up after surgery. In order to avoid the intra-observer bias, all radiological parameters were measured using picture archiving and communication systems (PACS) by 2 attending spinal surgeons who were not involved in the surgery, and the average value of their measurements was used for analysis.

The parameters examined in the pre- and postoperative posteroanterior standing full-length radiographs respectively included coronal Cobb angles of the main thoracic coronal curve, Risser sign, UIV and LIV.

Sagittal parameters were measured on lateral standing radiographs:C2–C7 angle, the Cobb angle between the lower endplate of C2 and C7; thoracic inlet angle (TIA), the angle between the vertical line of the T1 superior endplate and the line connecting the midpoint of the T1 superior endplate to the upper end of the sternum; T1 slope, the angle between the horizontal and the T1 superior endplate; neck tilt (NT), the angle between the plumb line and the line connecting the midpoint of the T1 superior endplate to the upper end of the sternum; C2-7 sagittal vertical axis (C2-7 SVA), the horizontal distance between the C2 plumb line and the posterior corner of C7; proximal thoracic kyphosis (PTK), the Cobb angle between the upper endplate of T1 and the upper endplate of T5; thoracic kyphosis (TK), the Cobb angle between the upper endplate of T5 and the lower endplate of T12; lumbar lordosis (LL), the Cobb angle between the upper endplate of L1 and S1. Negative values indicated lordosis while positive values indicated kyphosis.

All data were analyzed by using SPSS software (version 22.0; IBM Corp., Armonk, NY, USA). All values are presented as mean ± standard deviation. Quantitative data were analyzed by using Student’s t test or Mann–Whitney U test as appropriate (including all coronal and sagittal parameters except sex, UIV or LIV). Categorical data were analyzed by using the *χ* ^2^ test or Fisher’s exact test (including sex, UIV and LIV). The one-way ANOVA test was also utilized to evaluate the parameters among different groups. The relationships between variables were performed using Pearson’s correlation test. Receiver operation characteristic (ROC) curves and calculation of area under the curve (AUC) were used to estimate which TIA could be used to predict the existence of CK before and after the surgery. Statistical significance was set at *P* < 0.05.

## Results

A total of 50 patients with Lenke 1 AIS (10 males and 40 females) were enrolled in this study. The mean patient age at surgery was 15.20 ± 3.83 years. The mean Risser was 3.04 ± 1.62. The mean Cobb angle of the thoracic curve was 52.0° ± 9.1° (range: 40.0–76.0°). The mean follow-up period was 20.3 ± 12.7 months (range: 12–58 months).

There were 16 (32.0%) patients with CL and 34 (68.0%) patients with CK. The comparison of parameters between the patients with CL and CK was listed in Table 1. No significant differences were found in age, sex, Risser sign, Cobb or C2-7 SVA (Table 1). However, the TIA, T1 slope, PTK, TK and LL of patients with cervical lordosis (CL) were significantly greater than that of patients with CK (*P* < 0.05). The TIA of patients with CL and CK was 76.3 and 63.0°, respectively. And the ROC curve shown that the cutoff value was 71°. The Pearson correlation analysis shown that the preoperative C2-7 angle correlated significantly with TIA (*r* = −0.635), T1 slope (*r* = −0.758), C2-7 SVA (*r* = 0.373), PTK (*r* = −0.437) and TK (−0.463) (Table 2).

**Table 1:** Preoperative parameters between the patients with CL and CK (*n* = 50)

	CL (*n* = 16)	CK (*n* = 34)	*P*
Age	16.7 ± 5.6	14.5 ± 2.5	0.059
Sex	/	/	0.256
Males	5	5	
Females	11	29	
Risser	3.3 ± 2.1	2.9 ± 1.4	0.598
Cobb (°)	54.0 ± 11.9	50.4 ± 9.2	0.259
C2-7 angle (°)^a^	−16.0 ± 10.4	12.9 ± 10.1	0.000
TIA (°)^a^	76.3 ± 14.2	63.0 ± 7.8	0.002
C2-7 SVA (cm)	1.5 ± 1.2	2.0 ± 0.7	0.136
T1 slope (°)^a^	21.3 ± 10.4	8.7 ± 6.9	0.000
PTK (°)^a^	13.1 ± 9.3	4.6 ± 8.1	0.002
TK (°)^a^	32.5 ± 20.8	20.77 ± 11.8	0.047
LL (°)^a^	−62.4 ± 15.3	−49.0 ± 12.6	0.002

Values indicate mean ± standard deviation unless otherwise specified

*CL* cervical lordosis, *CK* cervical kyphosis, *AIS* adolescent idiopathic scoliosis, *TIA* thoracic inlet angle, *SVA* sagittal vertical axis, *PTK* proximal thoracic kyphosis, *TK* thoracic kyphosis, *LL* lumbar lordosis

^a^Significant difference

**Table 2:** Correlations of the preoperative parameters (*n* =50)

		C2-7 angle	TIA	T1 slope	C2-7 SVA	PTK	TK	Cobb
C2-7 angle	*r*	/	−0.635^a^	−0.758^a^	0.373^a^	−0.437^a^	−0.463^a^	−0.133
	*P*	/	0.000	0.000	0.008	0.001	0.001	0.359
TIA	*r*	−0.635^a^	/	0.715^a^	−0.258	0.126	0.583^a^	0.362^a^
	*P*	0.000	/	0.000	0.070	0.381	0.000	0.010
T1 slope	*r*	−0.758^a^	0.715^a^	/	−0.074	0.438^a^	0.660^a^	0.349^a^
	*P*	0.000	0.000	/	0.608	0.001	0.000	0.013
C2-7 SVA	*r*	0.373^a^	−0.258	−0.074	/	0.113	−0.104	0.054
	*P*	0.008	0.070	0.608	/	0.433	0.472	0.708
PTK	*r*	−0.437^a^	0.126	0.438^a^	0.113	/	−0.235	−0.335^a^
	*P*	0.001	0.381	0.001	0.433	/	0.100	0.018
TK	*r*	−0.463^a^	0.583^a^	0.660^a^	−0.104	−0.235	/	0.604^a^
	*P*	0.001	0.000	0.000	0.472	0.100	/	0.000
Cobb	*r*	−0.133	0.362^a^	0.349^a^	0.054	−0.335^a^	0.604^a^	/
	*P*	0.359	0.010	0.013	0.708	0.018	0.000	/

*AIS* adolescent idiopathic scoliosis, *TIA* thoracic inlet angle, *SVA* sagittal vertical axis, *PTK* proximal thoracic kyphosis, *TK* thoracic kyphosis, *LL* lumbar lordosis

^a^Significant difference

The mean pre- and postoperative values of the coronal and sagittal radiographical parameters in all Lenke 1 patients with AIS were presented in Table 3. Significant differences could be seen for Cobb angle, T1 slope, PTK and TK (*P* < 0.05).In contrast, no significant differences were detected for C2-7 angle, TIA, C2-7 SVA and LL.

**Table 3:** Details of pre- and postoperative radiologic findings in all patients (*n* = 50)

	Preop	Postop	*P*
C2-7 angle (°)	3.6 ± 17.0	0.3 ± 16.5	0.111
TIA (°)	67.3 ± 11.9	67.4 ± 11.7	0.869
T1 slope (°)^a^	12.7 ± 10.0	15.9 ± 8.9	0.005
C2-7 SVA (cm)	1.8 ± 0.9	1.9 ± 0.9	0.641
PTK (°)^a^	7.3 ± 10.0	14.2 ± 10.4	0.000
TK (°)^a^	24.5 ± 16.0	15.6 ± 5.4	0.000
LL (°)	−53.3 ± 14.8	−49.7 ± 9.9	0.099
Cobb (°)^a^	51.6 ± 10.2	7.7 ± 5.6	0.000

Values indicate mean ± standard deviation unless otherwise specified

*AIS* adolescent idiopathic scoliosis, *Preop* preoperation, *Postop* postoperation, *TIA* thoracic inlet angle, *SVA* sagittal vertical axis, *PTK* proximal thoracic kyphosis, *TK* thoracic kyphosis, *LL* lumbar lordosis

^a^Significant difference

Table 4 shows the pre- and postoperative values of the coronal and sagittal radiographical parameters in the 4 subgroups of patients according the change of cervical curvature after surgery. In patients with postoperative CL, who had preoperative CK, there was significant reduction in TK (26.3 vs. 16.5), while significant increase in PTK (2.6 vs. 16.8). The patients who had preoperative CL and remained lordotic postoperatively, also had significant decrease in TK (38.8 vs. 18.9) and significant increase in PTK (13.6 vs. 28.9).In patients who had preoperative CL and had CK postoperatively, both the PTK and TK were significantly decreased after surgery (12.4 vs. 8.5, 24.5 vs. 12.8, respectively). However, in patients with uncorrected CK after operation, the PTK was not significantly changed after surgery and their TK shown a significant decrease from 18.1 to 14.8°.

**Table 4:** Pre- and postoperative parameters in the 4 subgroups

	Preop CL-Postop CL (*n* = 9)			Preop CK-Postop CL (*n* = 11)			Preop CL-Postop CK (*n* = 7)			Preop CK-Postop CK (*n* = 23)		
	preop	postop	*P*	preop	postop	*P*	preop	postop	*P*	preop	postop	*P*
C2-7 angle (°)	−18.9 ± 11.4	−24.9 ± 13.6	0.158	7.6 ± 7.3	−7.0 ± 8.5	0.001^a^	−12.4 ± 8.3	5.9 ± 4.5	0.001^a^	15.3 ± 10.5	11.9 ± 8.0	0.173
TIA (°)	82.5 ± 14.5	82.8 ± 14.6	0.783	66.9 ± 7.4	67.9 ± 7.0	0.578	68.5 ± 9.7	65.9 ± 10.7	0.012^a^	61.1 ± 7.3	61.5 ± 6.1	0.707
T1 slope (°)	25.0 ± 12.2	29.1 ± 7.6	0.193	11.0 ± 7.5	16.8 ± 6.7	0.016^a^	16.7 ± 5.1	14.0 ± 2.8	0.057	7.6 ± 6.5	11.0 ± 5.8	0.056
C2-7 SVA (cm)	1.7 ± 1.6	1.7 ± 1.6	0.942	1.9 ± 0.9	1.6 ± 0.8	0.255	1.3 ± 0.4	1.7 ± 0.4	0.075	2.0 ± 0.6	2.1 ± 0.6	0.378
PTK (°)	13.6 ± 11.8	28.9 ± 10.4	0.001^a^	2.6 ± 8.1	16.8 ± 6.6	0.000^a^	12.4 ± 5.5	8.5 ± 3.1	0.040^a^	5.5 ± 8.2	9.0 ± 6.9	0.052
TK (°)	38.8 ± 24.0	18.9 ± 4.1	0.044^a^	26.3 ± 16.4	16.5 ± 4.7	0.031^a^	24.5 ± 13.2	12.8 ± 3.0	0.045^a^	18.1 ± 8.0	14.8 ± 6.2	0.043^a^
LL (°)	−65.3 ± 18.0	−47.1 ± 7.3	0.008^a^	−56.2 ± 14.5	−50.4 ± 8.5	0.106	−58.7 ± 11.3	−53.5 ± 13.7	0.462	−45.6 ± 10.3	−49.2 ± 10.3	0.157
Cobb (°)	59.3 ± 12.3	10.6 ± 5.1	0.000^a^	50.2 ± 12.1	9.0 ± 8.9	0.000	47.0 ± 7.2	6.1 ± 2.8	0.000^a^	50.6 ± 7.8	6.4 ± 3.8	0.000^a^

Values indicate mean ± standard deviation unless otherwise specified

*Preop* preoperation, *Postop* postoperation, *CL* cervical lordosis, *CK* cervical kyphosis, *TIA* thoracic inlet angle, *SVA* sagittal vertical axis, *PTK* proximal thoracic kyphosis, *TK* thoracic kyphosis, *LL* lumbar lordosis

^a^Significant difference

The Pearson correlation analysis shown that the postoperative C2-7 angle correlated significantly with the TIA (*r* = −0.613), T1 slope (*r* = −0.840), PTK (*r* = −0.698) and TK (−0.447) (Table 5). The TIA of patients with CK after surgery was significantly smaller than that of patients with CL postoperatively (62.5° ± 7.4° vs. 74.6° ± 13.2°, *p* < 0.05). And the ROC curve had shown that the cutoff value was 62°.

**Table 5:** Correlations of the postoperative parameters (*n* =50)

		C2-7 angle	TIA	T1 slope	C2-7 SVA	PTK	TK	Cobb
C2-7 angle	*r*	/	−0.613^a^	−0.840^a^	0.191	−0.698^a^	−0.447^a^	−0.279^a^
	*P*	/	0.000	0.000	0.185	0.000	0.001	0.049
TIA	*r*	−0.613^a^	/	0.692^a^	−0.075	0.544^a^	0.324^a^	0.274
	*P*	0.000	/	0.000	0.606	0.000	0.022	0.054
T1 slope	*r*	−0.840^a^	0.692^a^	/	0.159	0.796^a^	0.423^a^	0.300^a^
	*P*	0.000	0.000		0.269	0.000	0.002	0.034
	*N*	50	50	50	50	50	50	50
C2-7 SVA	*r*	0.191	−0.075	0.159	/	0.293^a^	−0.129	−0.097
	*P*	0.185	0.606	0.269	/	0.039	0.370	0.503
PTK	*r*	−0.698^a^	0.544^a^	0.796^a^	0.293^a^	/	0.128	0.057
	*P*	0.000	0.000	0.000	0.039	/	0.378	0.692
TK	*r*	−0.447^a^	0.324^a^	0.423^a^	−0.129	0.128	/	0.453^a^
	*P*	0.001	0.022	0.002	0.370	0.378	/	0.001
Cobb	*r*	−0.279^a^	0.274	0.300^a^	−0.097	0.057	0.453^a^	/
	*P*	0.049	0.054	0.034	0.503	0.692	0.001	/

*AIS* adolescent idiopathic scoliosis, *TIA* thoracic inlet angle, *SVA* sagittal vertical axis, *PTK* proximal thoracic kyphosis, *TK* thoracic kyphosis, *LL* lumbar lordosis

^a^Significant difference

The UIV was in T2–T5 in this study and there were 6 (12.0%), 28 (56.0%), 13 (26.0%) and 3 (6.0%) patients with T2, T3, T4 and T5, respectively. The one-way ANOVA test shown that the pre- and postoperative values of C2-7 angle were similar among the groups classified according to the UIV level (T2-T5).

## Discussion

The incidence of CK in AIS patients was 40–86% [6, 13, 17]. Our CK incidence of 68%(34/50) was comparable with the previous studies. Upsani et al. [1] and Hiyama A et al. [13] have reported that the preoperative TK of patients with AIS was smaller than that in nonscoliotic controls. They considered the reason may be that the TK may be smaller when seen from a true lateral view at the apex because of vertebral rotation and wedging in patients with a main thoracic curve. Moreover, Ye F et al. [12] and Hwang et al. [18] found that the TK of patients with CK was smaller than that of patients with CL. Our analysis revealed that the TK of patients with CK was significantly smaller than that of patients with CL preoperatively. And Pearson correlation analysis shown that the preoperative CK correlate significantly with preoperative TK (*r* = −0.463) in the present study.

Another important parameter that may influence cervical alignment was the amount of T1 slope [12, 15, 16, 19, 20]. Pesenti S et al. [16] regarded T1 as a valuable assessment of cervicothoracic alignment. As a primary parameter of T1, the T1 slope will determine the amount of subaxial lordosis required to maintain the center of gravity of the head in a balanced position, and it will vary depending on global spinal alignment [7]. In our study, the T1 slope of patients with CK was significantly smaller than that of patients with CL. And Pearson correlation analysis shown that the preoperative C2-7 angle correlated significantly with preoperative T1 slope (*r* = −0.758).It was worth mentioning that there are 5 patients whose T1 slope were less than 0° in the present study, all of them were patients with cervical kyphosis.

Both TK and T1 slope mentioned above were not constant parameter, they could be influenced by posture. That means, TK or T1 slope cannot be used as a predicting parameter for C2-7 angle if the patient was not in standing position. Lee et al. [21] introduced the concepts of neck tilt (NT) and thoracic inlet angle (TIA). The NT was defined as the angle between the plumb line and the line connecting the midpoint of the T1 superior endplate to the upper end of the sternum. The TIA was defined as the angle between the vertical line of the T1 superior endplate and the line connecting the midpoint of the T1 superior endplate to the upper end of the sternum. Thoracic inlet (TI) is a bony circle without range of motion, which is composed of the T1 vertebral body, the first ribs on both sides and the upper part of the sternum [22]. TIA was a constant morphological parameter and was not changed by the position or under any conditions, like pelvic incidence (PI) in the spino-pelvic unit. Geometrically, a formula, “TIA = T1 slope + NT” was formed similar to “PI = sacral slope (SS) + pelvic tilt (PT)”. Lee et al. [21] indicated that the sagittal balance of the cranium and cervical spine could be influenced by the shape and orientation of TI to get a balanced upright posture and horizontal gaze. Furthermore, they believe that large TIA increases T1 slope and finally increase CL to obtain a horizontal gaze and sagittal alignment of cervical spine with minimum energy expenditure. In contrast, a small TIA will first lead to a smaller T1 slope, and then cause a decrease in the CL, which finally leads to a cervical kyphosis. In this study, the TIA of all 50 patients was not changed after the surgery (67.2° vs. 67.4°). And we found that the TIA of patients with CK was significantly smaller than that of patients with CL (63.0° vs. 76.3°). The ROC curve shown that the patients whose TIA less than 71° were more likely to have CK. Moreover, the preoperative TIA was significantly correlated with T1 slope (*r* = 0.715) and C2-7 angle (*r* = −0.635).

After the surgery, there are still 60% (30/50) of patients with cervical kyphosis while 40%(20/50) of them with cervical lordosis. And we noted that the TIA of patients with CK after surgery was significantly smaller than that of patients with CL postoperatively (62.5° vs. 74.6°). The ROC curve shown that the cutoff value of the TIA was 62°. Thus, we suggested that the TIA in Lenke 1 AIS patients could be a good indicator of CK after surgery: The Lenke 1 AIS patients with TIA less than 62° would lead to the postoperative uncorrected or new onset of CK.

Quan GM et al. [23] found that the amount of curve correction in the coronal plane and the reduction of kyphosis in the thoracic sagittal profile had a strong positive correlation in patients with AIS undergoing pedicle screw instrumentation. Both pedicle screws and hybrid instrumentation (hooks and pedicle screws) have a hypokyphotic effect on the thoracic spine, which could lead to the occurrence of CK after the surgery [14, 18]. In this study, all 50 patients underwent surgical treatment with 1-stage posterior pedicle-screw correction and fusion. And their TK were significantly decreased after surgery.

**Fig. 1:**
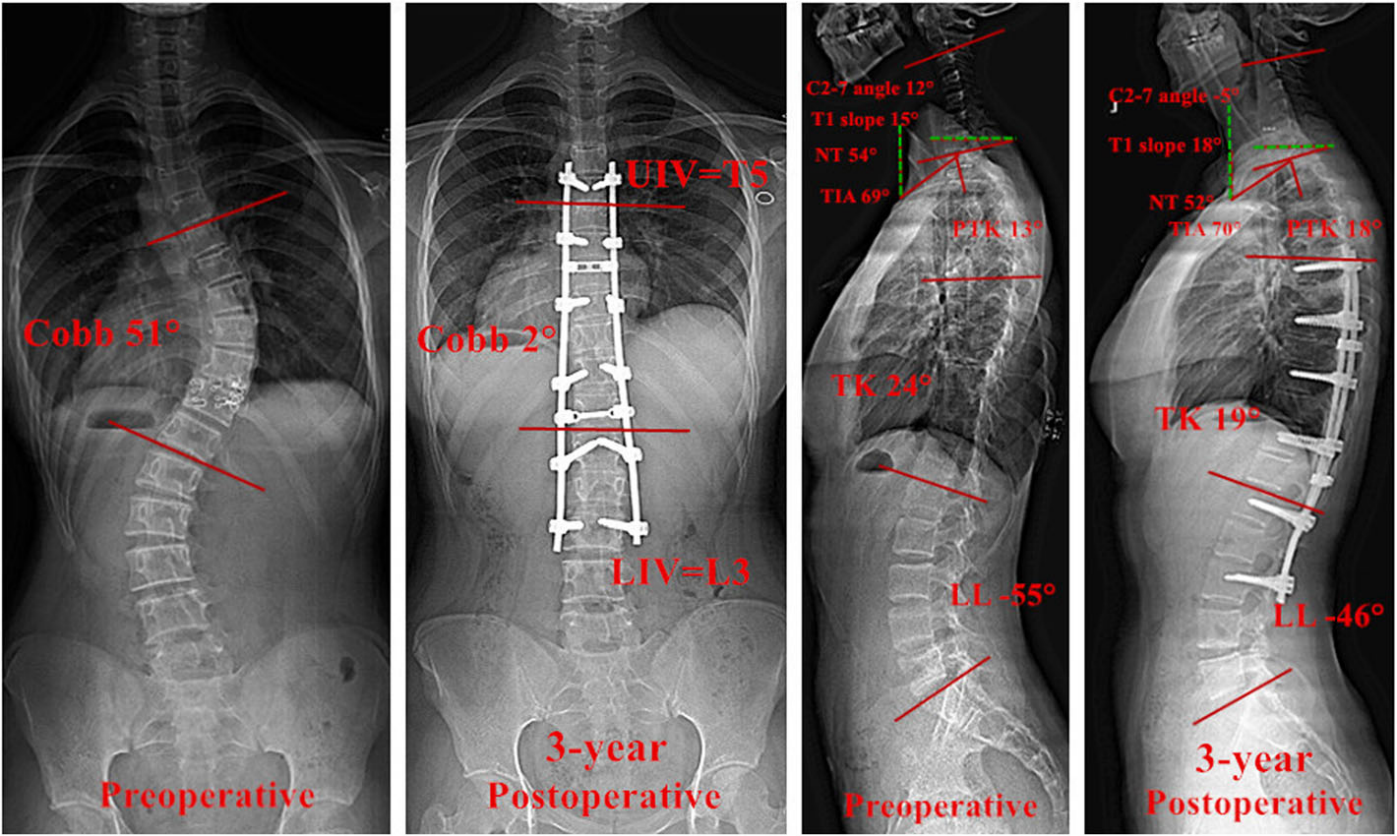
The pre- and 3-year postoperative radiographs of a Lenke 1 patient with AIS who had preoperative CK and postoperative CL. The coronal Cobb angle of the *main curve* was corrected from 51° preoperative to 2° postoperative (the rate of corrective Cobb angle was 96.1%). The C2-7 angle varied from 12° preoperative to −5° postoperative. The PTK increased from 13° preoperative to 18° postoperative. The TK decreased from 24° preoperative to 19° postoperative. AIS, adolescent idiopathic scoliosis; CK, cervical kyphosis; CL, cervical lordosis; PTK, proximal thoracic kyphosis; TK, thoracic kyphosis

**Fig. 2:**
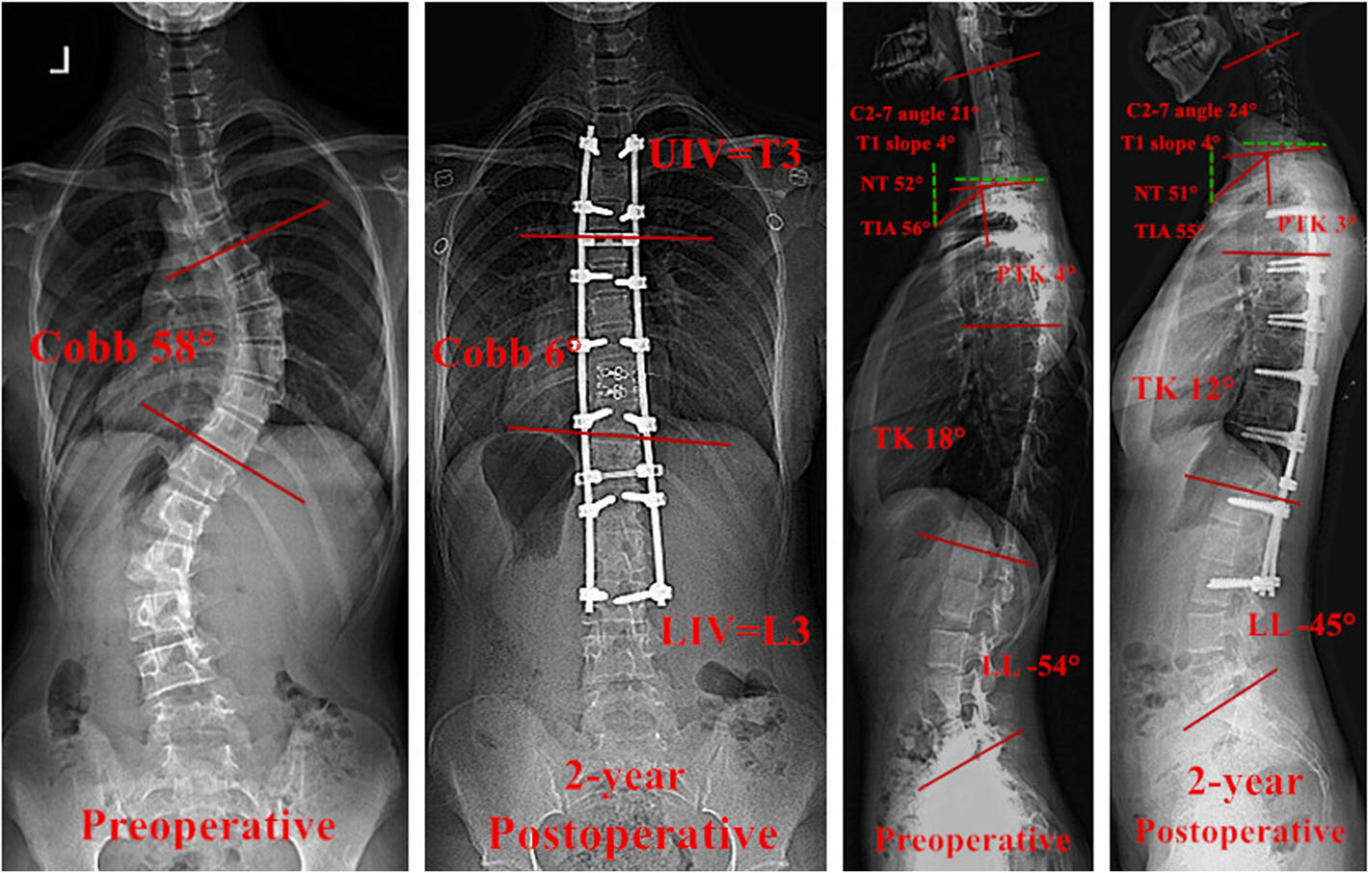
The pre- and 2-year postoperative radiographs of a Lenke 1 patient with AIS who had uncorrected CK after surgery. The coronal Cobb angle of the *main curve* was corrected from 58° preoperative to 6° postoperative (the rate of corrective Cobb angle was 89.7%). The C2-7 angle varied from 21° preoperative to 24° postoperative. The PTK varied from 4° preoperative to 3° postoperative. The TK decreased from 18° preoperative to 12° postoperative. AIS, adolescent idiopathic scoliosis; CK, cervical kyphosis; CL, cervical lordosis; PTK, proximal thoracic kyphosis; TK, thoracic kyphosis

Charles et al. [24] have found that a postoperative increase of PTK correlated with a compensatory increased T1 slope, which could trigger cervical lordosis. In our study, the postoperative PTK correlated significantly with postoperative T1 slope (*r* = 0.796) and C2-7 angle (*r* = −0.698). In patients with postoperative cervical lordosis (CL), there was a significant increase in their PTK and a reduction in their TK (Fig. 1), regardless of preoperative CL or CK. In patients whose CL deteriorated to CK after surgery, both their PTK and TK significantly decreased after surgery. It indicated that the increased PTK after the operation could have a beneficial effect on the improvement of CL. However, in patients with uncorrected CK after operation, the PTK did not significantly vary after surgery (Fig. 2).

The relation between the upper instrumented vertebra (UIV) level and cervical alignment has been evaluated in some studies [14, 15, 18]. Legarreta et al. [14] found that cervical kyphosis induced by scoliosis correction was correlated with the level of the UIV, especially in cases in which the UIV is above the T4 level. However, Yanik et al. [15] indicated that the postoperative CK was independent from UIV level. In this study, the pre- and postoperative values of C2-7 angle were similar among the groups classified according to the UIV level (T2-T5), which meant the UIV level was not associated with CK. For one thing, UIV was much more determined by preoperative coronal curves instead of parameters in sagittal malalignment. For another, the restoration of the PTK mainly relies on the surgeon’s experience rather than the aid of UIV.

Several limitations still exist. First, this was a retrospective study and lacked longer term follow-up time. Therefore, future prospective and longitudinal studies with larger numbers of patients and longer follow-up period until skeletal maturity are needed to evaluate the cervical alignment in patients with AIS. Second, our study included only radiological data and no functional scores were used to evaluate the patients’ clinical outcomes. So, future studies should focus on the investigation of the correlation between CK and clinical outcomes.

## Conclusions

The incidence of CK in Lenke type 1 AIS patients is high (68%), especially in those who have a TIA less than 71°. A small TIA will first lead to a smaller T1 slope, and then cause a decrease in the CL, which finally leads to a cervical kyphosis. The TIA should be a good indicator of CK after surgery: patients with TIA less than 62° would lead to the postoperative uncorrected or new onset of CK. The increased PTK after the operation could have a beneficial effect on the improvement of CL. However, it seems that the UIV level was not associated with CK.

## Abbreviations

AIS: Adolescent idiopathic scoliosis
AUC: Area under the curve
CK: Cervical kyphosis
CL: Cervical lordosis
HRQOL: Health-related quality of life
LIV: Lower instrumented vertebra
LL: Lumbar lordosis
NT: Neck tilt
PACS: Picture archiving and communication systems
PI: Pelvic incidence
PT: Pelvic tilt
PTK: Proximal thoracic kyphosis
ROC: Receiver operation characteristic
SS: Sacral slope
SVA: Sagittal vertical axis
TIA: Thoracic inlet angle
TK: Thoracic kyphosis
UIV: Upper instrumented vertebra
